# Distinct migration patterns of adult neural stem cells derived from hippocampal and ventricular niches

**DOI:** 10.3389/fnagi.2026.1727458

**Published:** 2026-06-03

**Authors:** Sarnai Amartumur, Huong Nguyen, Trung Hoang, Thuy Huynh, Chaejeong Heo

**Affiliations:** Department of Biophysics, Institute of Quantum Biophysics, Sungkyunkwan University, Suwon, Republic of Korea

**Keywords:** adult neural stem cells niche, bilayer spheroids, microfluidic chip, migration, neurogenesis

## Abstract

Adult neurogenesis occurs in the two primary areas of the human brain, namely the subgranular zone (SGZ) of the hippocampus and the subventricular zone (SVZ) of the lateral ventricles. Neural stem cells (NSCs) from these niches generate new neurons and glia that contribute to plasticity and repair; however, their niche-associated cellular behaviors remain poorly characterized due to limited access to human brain tissue and the lack of physiologically relevant *in vitro* models. Here, we comparatively investigated the morphological features, migration behaviors, and neural-glia organization of NSCs derived from the SGZ and SVZ of the adult mouse brain using microfluidic-based cultures. SVZ-derived NSCs exhibited longer neurites, greater motility, and dispersed migration patterns, whereas SGZ-derived NSCs displayed shorter neurites, reduced motility, and collective, radial migration. In bilayer spheroid cultures, SGZ progenitors retained higher proportions of TUJ1^+^ and GFAP^+^ cells in the inner core over time, reflecting compact local integration characteristic of the dentate gyrus (DG), whereas SVZ progenitors dispersed outward, with fewer cells retained centrally, consistent with tangential migration along the rostral migratory stream. Exposure of DG–hippocampal spheroids to oligomeric amyloid-*β* (Aβ), followed by morphological assessments and targeted gene-expression profiling, showed disruption of spheroid organization along with enrichment of neuroinflammatory- and apoptosis-related gene signatures, suggesting that the model reflects selected features of Aβ-induced neurotoxicity. Together, these findings characterize niche-associated differences in the migration dynamics of adult NSCs *in vitro* and suggest an engineered platform for future mechanistic studies and disease modeling.

## Introduction

Neurogenesis occurs throughout embryonic development and continues into adulthood in mammals, comprising a sequential cascade of cellular events including proliferation, differentiation, and migration. Particularly, adult neurogenesis has emerged as a key area of interest in neural stem cell (NSC)-based regenerative strategies, as it contributes to neuronal plasticity, learning, memory, mood response, and repair (Brunet et al., 2023). Although its decline with aging remains debated, adult neurogenesis is well established in two primary brain regions: the subventricular zone (SVZ) of the lateral ventricles and the subgranular zone (SGZ) of the hippocampus, with other potential sites still under discussion (Bernier et al., 2002; Zhao et al., 2003).

The SVZ, lining the walls of the lateral ventricles in the adult human brain, is one of the primary niches of NSCs. SVZ-derived NSCs generate multi-lineage brain cells, including neurons, astrocytes, and oligodendrocytes, and they contribute to brain plasticity by providing newly born cells to regions of the brain where they are needed. In rodents, these newly generated cells migrate from the SVZ to the olfactory bulb through the well-known rostral migratory stream. In the human brain, migration patterns are less well defined but are considered to contribute to striatal and cortical regions and participate in tissue repair and circuit development. The dentate gyrus (DG), a hippocampal subregion implicated in learning and memory in mammals, contains the SGZ, a neurogenic niche in which resident NSCs generate new neurons through a process termed adult hippocampal neurogenesis (AHN). DG-derived cells migrate over a shorter distance, where they incorporate into the granule cell layer of the DG within the hippocampal circuitry and participate in neural plasticity and cognitive function.

With aging, neurogenesis is reduced due to several changes, including decreased proliferative capacity, alteration in the niche caused by aging-related vascular changes, and extracellular signaling deficiency (Kuhn et al., 1996; Ben Abdallah et al., 2010; Encinas et al., 2011). Understanding AHN, particularly cellular behavior, is an important objective for discovering human neural repair and developing regenerative treatment for aging-related neurological diseases. Moreover, the hippocampal area and its SGZ neurogenic niches are highly vulnerable in Alzheimer’s disease (AD) (Salta et al., 2023). Evidence indicates that amyloid-*β* (Aβ), Tau deposits, and neuroinflammation impair NSC proliferation, differentiation, and neuronal integration, leading to reduced neurogenesis and cognitive decline in AD. Consequently, modeling SGZ neurogenesis provides critical insight into both the pathogenesis of AD and the potential of hippocampal stem cell-based regenerative therapies.

However, detailed characterization of adult neurogenic cell populations has been constrained by the limited accessibility of adult human brain tissue, although several studies have demonstrated distinct histological and single-cell profiling features when comparing AHN in humans, macaques, and mice (Zhou et al., 2025). Moreover, most *in vitro* models of AHN focus on NSC proliferation and differentiation, whereas comparative studies investigating the cellular characteristics of the two neurogenic niches, especially migratory patterns, remain limited. In addition, current models often rely on embryonic neurons or induced pluripotent stem cell (iPSC)-derived neurons, which are limited in their ability to recapitulate the adult-specific characteristics, particularly in terms of migration patterns (Yu et al., 2014).

Here, we report distinct differences between neurogenic niches in morphological and migratory behavior using microfluidic chip devices. Taking advantage of compartmentalized microchannels, we were able to observe distinct niche-specific migratory motility. Moreover, bilayer spheroid formation allows *in vitro* characterization of SVZ- and SGZ-derived cells, including migration, neural-glia distribution, and dynamic changes over an adult-cell scaffold. We believe that, by combining an engineering-controlled microenvironment, our results offer comparative insights into niche-associated differences in adult NSC behavior that may be further explored through quantitative studies for NSC-based therapies and neurological diseases.

## Materials and methods

### Cell culture

The SVZ- and SGZ-derived stem cells were extracted from 12-week-old male C57BL/6 mice (Saeronbio Inc., Uiwang-si, South Korea). Animal procedures were performed in accordance with guidelines approved by the Institutional Animal Care and Use Committee (IACUC) of Sungkyunkwan University School of Medicine (SUSM). Briefly, tissue extracted from the lateral ventricle and hippocampal area was chopped, digested with Accutase (Gibco, United States), and cultured in proliferation medium consisting of NeuroCult^™^ Basal Medium supplemented with 10% NeuroCult^™^ Proliferation Supplement (Mouse & Rat) (STEMCELL Technologies, Canada), recombinant human epidermal growth factor (20 ng/mL, STEMCELL Technologies), recombinant human basic fibroblast growth factor (10 ng/mL, STEMCELL Technologies), heparin (2 μg/mL, STEMCELL Technologies), and penicillin/streptomycin (100 IU/mL, Gibco). Neurospheres reached a diameter of ~100–150 μm after 5–7 days of culture. Passaged neurospheres were subsequently used for experiments after being dissociated with Accutase. The stem-cell identity of NSCs was confirmed by immunostaining, as evidenced by positive expression of SOX2 and GFAP (Supplementary Figure 1A).

Adult mouse hippocampal neurons gifted from Prof. Park’s group were maintained in high-glucose Dulbecco’s modified Eagle medium (DMEM; Sigma-Aldrich, United States) supplemented with 10% fetal bovine serum (Gibco) and penicillin/streptomycin (100 IU/mL; Thermo Fisher Scientific, United States). Cultures were kept in a humidified incubator at 37 °C with 5% CO₂ and passaged according to standard procedures once they reached approximately 80% confluency. For chip-based experiments, confluent cells were rinsed with Dulbecco’s phosphate-buffered saline, dissociated using 0.25% trypsin–EDTA (Gibco), and collected by centrifugation. The resulting pellet was resuspended in fresh medium at a density of 2.5 × 10^6^ or 5 × 10^6^ cells/mL. The neuronal identity and synaptic maturity of adult hippocampal neurons were confirmed by positive expression of MAP2, NeuN, and PSD95 with negative GFAP expression (Supplementary Figure 1B).

### Microfluidic chip fabrication

The migration chip was fabricated via a soft photolithography technique. A silicon wafer was used to fabricate the master mold using a photosensitive polymer (SU8, Microchem) to generate a dual-layer master mold. The first layer consisted of an array of parallel microchannels with a height of 5 μm, a width of 10 μm, and a length of 600 μm, with an interchannel spacing of 15 μm. These microchannels connected two main chambers defined in the second layer with dimensions of 10 mm × 5 mm × 100 μm. After post-treatment, the master mold was replicated onto polydimethylsiloxane (PDMS) (SYLGARD 184) prepared by mixing at a 10:1 ratio and degassed for 30 min. The PDMS replicates were cured in an oven at 80 °C for 3 h. The cured PDMS replica was then detached, and inlet and outlet holes were punched with 1.5 mm biopsy punches. A confocal dish (SPL Sciences, United States) was used as the bottom surface, which was bonded by oxygen plasma. Before use in culture, the chip device was sterilized under UV for 30 min. The chips were treated with poly-D-lysine solution (10 μg/mL, A38904, Thermo Fisher Scientific) for 2 h at room temperature, then washed several times with PBS (Supplementary Figures 4A,C).

The spheroid microfluidic chip was designed in SolidWorks (Dassault Systems, United States) and fabricated using a stereolithography three-dimensional (3D) printer (Form 2, Formlabs, United States) at 25 μm resolution. Printed molds were washed in isopropyl alcohol for 1 h, dried at 80 °C for 1 h, and sputter-coated with platinum. Before PDMS casting, molds were silanized with trichloro(1H,1H,2H,2H-perfluorooctyl)silane in a vacuum desiccator for 30 min to render the surface hydrophobic. PDMS (SYLGARD 184, DOW Inc., United States) was mixed at a 10:1 base-to-curing agent ratio, poured onto molds, degassed, and cured at 80 °C for 3 h. The cured PDMS replica was peeled off, and 1.5 μm inlet/outlet holes were punched. For assembly, liquid PDMS was spin-coated on glass slides (500 rpm, 30 s) to bond the PDMS replica, followed by attachment of the top cover. The assembled chip was further cured at 80 °C for ≥3 h. A medium reservoir (15 × 25 mm) was bonded to the inlet to allow medium supply for cell culture (Supplementary Figures 3B,D).

### Neurospheres migration assay and chip-based assay

Neurospheres migration assays were performed following a previously described protocol with minor modifications (Masood et al., 2020). Briefly, neurospheres were placed on a laminin-coated dish (10 μg/mL, 23,017-015, Invitrogen) and incubated in differentiation medium for 24 h.

For the chip-based assay, a single-cell suspension of NSCs at a concentration of 1 × 10^7^ cells/mL in NeuroCult^™^ Basal Medium and 10% NeuroCult^™^ Differentiation Supplement (Mouse & Rat) (STEMCELL Technologies) was prepared. A total volume of 5 μL was seeded into one side of the main chamber. Before seeding, cells were stained with Hoechst 33342 (Thermo Fisher Scientific) for live imaging. The opposite chamber was replenished with medium to maintain intrinsic motility under compartmentalized static conditions. Cells were allowed to attach, and migration was observed within the experimental period. The migration chip was imaged in real time using a humidified live chamber (Live Cell Instrument, South Korea) maintained at 37 °C and 5% CO_2_ using a Leica DMi8 microscope (Leica Microsystems, Germany). Image acquisition was conducted under minimized exposure time to reduce phototoxicity. No morphological abnormalities or overt nuclear fragmentation were observed during the imaging period.

### Bilayer spheroid culture and live-cell labeling

Microfluidic chips were sterilized with 500 μg/mL gentamycin (Gibco) for 4 h and coated with 2% Pluronic F-127 in PBS (24 h) to minimize nonspecific adhesion. DG single cells (2.5 × 10^6^ cells/mL) in NeuroCult^™^ Basal Medium with 10% Differentiation Supplement (STEMCELL Technologies) were loaded into the cell inlet. Cell loading was achieved via gravity-driven flow by introducing 300 μL of cell suspension into the inlet and gently aspirating from the cell outlet, allowing cells to accumulate within the spheroid chamber and self-assemble into neurospheroids within 24 h. Subsequently, 300 μL of hippocampal cells (1.25 × 10^6^ cells/mL) were introduced by hydrostatic pressure into the first core spheroid through the cell inlet and allowed to settle for 30 min before initiating medium flow. Cultures were maintained at 37 °C with 5% CO₂ under gravity-driven medium flow, with daily medium replacement. All procedures were identical for both SGZ- and SVZ-derived bilayer spheroid formation. For distribution analysis, the outer region of the bilayer spheroid was defined as a peripheral ring corresponding to 10% of the total spheroid diameter measured inward from the boundary. The inner core was defined as the remaining central area.

### Immunostaining

Bilayer spheroids were collected on days 2, 3, and 5, fixed in 4% paraformaldehyde at 4 °C overnight, permeabilized with 0.3% Triton X-100, and blocked with serum. Samples were incubated with primary antibodies overnight at 37 °C, followed by secondary antibodies for 4 h at room temperature. Nuclei were counterstained with DAPI (1 μg/mL). Spheroids were mounted in confocal dishes with mounting solution (Binaree, South Korea). Image acquisition was performed using a Leica Thunder DMi8 microscope. The following primary antibodies were used: TUJ1 (1:750, Abcam), GFAP (1:150, Millipore), N-cadherin (1:500, Sigma-Aldrich), MAP2 (1:500, Abcam), NeuN (1:150, Abcam), SOX2 (1:500, Abcam), and PSD95 (1:250, Abcam). Phalloidin (iFlour647, Abcam) was used for 1 h at RT.

### Amyloid-*β* toxicity test and targeted gene-expression profiling of SGZ–hippocampal bilayer spheroids

A synthetic human Aβ_42_ peptide (AnaSpec, San Jose, CA, United States) was prepared in oligomeric form as described in a previous article (Heo et al., 2007). Lyophilized Aβ_42_ was reconstituted in 1% NH_4_OH to a stock concentration of 1 mg/mL and stored at −70 °C until use. For the experiment, oligomeric Aβ_42_ (oAβ_42_) was prepared by mixing Aβ_42_ stock solution (220 μM) with DMEM/F12 without phenol red and incubating the mixture at 4 °C for 24 h. The resulting stock was further diluted to 1 μM in culture medium. On day 1, DG spheroids were cultured with hippocampal cell suspensions that had been pretreated with oAβ_42_ for 1 day before seeding into the chip. Bilayer spheroids were then continuously exposed to 1 μM oAβ_42_-containing medium. Following treatment, spheroids were collected on day 5 for subsequent experiments. Total RNA was extracted from bilayer spheroids using the RNeasy Mini Kit (Qiagen, 74,104). Samples containing >100 ng RNA were analyzed using the nCounter Mouse Neuropathology Panel (NanoString Technologies, United States). Hybridization was performed with 5 μL RNA, 8 μL reporter probes, and 2 μL capture probes at 65 °C for 18 h, followed by processing on the nCounter Prep Station and scanning on the Digital Analyzer (280 fields). Data were normalized to internal reference genes using nSolver v4.0 (NanoString Technologies) and further analyzed in R software.

### Statistical analysis

Quantitative data are presented as mean ± standard error of the mean (SEM). Statistical comparisons between two groups were performed using two-tailed unpaired Student’s *t*-tests, whereas time-course comparisons were analyzed using two-way analysis of variance (ANOVA) in GraphPad Prism v.10.6.0 (GraphPad Software, United States). A *p*-value < 0.05 was considered statistically significant. Details of statistical analyses are provided in the corresponding figure legends.

## Results

### SVZ-derived cells exhibit higher motility and extended neurite outgrowth compared to SGZ-derived cells

**Figure 1:**
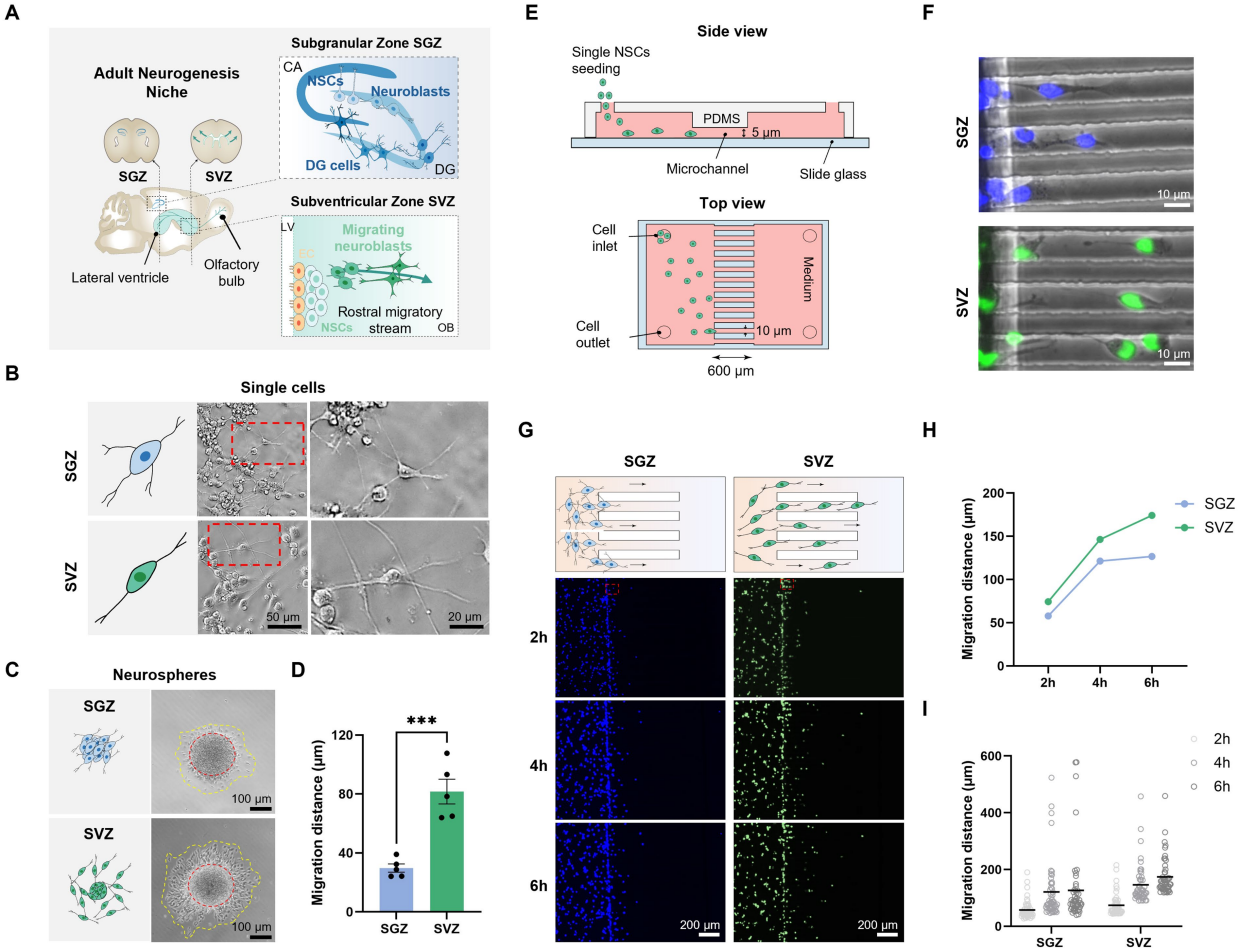
Differences in morphology and migration motility of adult neurogenesis niches in 2D conditions. **(A)** Schematic illustration comparing the adult mouse neurogenesis niches of hippocampal DG and the SVZ of the lateral ventricle. **(B)** Representative phase-contrast images of single SGZ- and SVZ-derived cells after 1 day of differentiation on laminin-coated substrates. Red dashed boxes show magnified views highlighting distinct cellular morphologies. Scale bars, 50 μm and 20 μm. **(C)** Distribution of differentiating cells migrating from SVZ- and SGZ-derived neurospheres *in vitro*. Red circles indicate neurosphere cores, and yellow lines mark the migration boundary. Scale bar, 100 μm. **(D)** Quantification of average migrating distance measured from the neurosphere edge to individual cells on day 1 (20 cells per neurosphere; *n* = 5 independent neurospheres per group). Data are presented as mean ± SEM. **(E)** Schematic illustration of the microfluidic chip used for the 2D migration assay. **(F)** Representative images of migrating cells within microchannels at the 2 h time point. Nuclei were stained with Hoechst and pseudo-colored blue (SGZ) and green (SVZ). Scale bar, 10 μm. **(G)** Time-lapse images of 2D migration within the microfluidic chip at 2, 4, and 6 h. Red dashed boxes indicate the regions magnified in **(F)**. (*n* = 1 chip). Scale bar, 200 μm. **(H)** Quantification of average migration distance over time within the microfluidic chip. **(I)** Scatter plot of individual cell migration distance at each time point (2, 4, and 6 h). Each dot represents a single cell (*n* = 50 per group). Statistical analysis in **(D)** was performed using an unpaired two-tailed Student’s *t*-test, ns, nonsignificant; *p < 0.05, **p < 0.01, and ***p < 0.001. CA, cornu ammonis; DG, dentate gyrus; EC, ependymal cells; LV, lateral ventricle; NSCs, neural stem cells; OB, olfactory bulb; SEM, standard error of the mean; SGZ, subgranular zone; SVZ, subventricular zone.

Adult neurogenesis occurs within two primary niches, the SGZ of the hippocampal DG and the SVZ of the lateral ventricles, which travel different distances to the targeted area (Figure 1A). To directly compare their properties, we cultured SGZ- and SVZ-derived NSCs in two-dimensional (2D) conditions. Morphological analysis revealed that SVZ-derived cells adopted an elongated bipolar shape with multiple extended neurites, whereas SGZ-derived cells were typically smaller with shorter, less branched processes (Figure 1B; Supplementary Figure 2). These morphological differences suggest that SGZ- and SVZ-derived NSCs possess distinct morphological identities, potentially reflecting differences in their respective native environments.

We further examined migration behavior using a neurospheres outgrowth assay similar to that described in a previous study (Masood et al., 2020) (Figures 1C,D). The result demonstrated niche-dependent behavior in which SGZ-derived cells displayed collective migration, whereas SVZ-derived cells dispersed more broadly and individually across the substrate (Figure 1C; Supplementary Figure 2). Quantification of migration distance confirmed significantly greater outgrowth in SVZ-derived cells compared to SGZ-derived cells (Figure 1D). To quantitatively assess intrinsic single-cell motility, we employed a microfluidic chip-based migration assay (Figures 1E,F). Both groups demonstrated progressive increases in motility over a 6 h period within the 600 μm microchannels (Figures 1F,G). In this single-chip time-course experiment, SVZ-derived cells appeared to migrate farther than SGZ-derived cells at each time point (Figure 1H). Additionally, considerable variability was observed in migration distances among both groups, especially among SGZ-derived cells that reached the distal end of the microchannels (Figure 1I). These findings suggest niche-associated differences in NSC morphology and migration behavior, consistent with their respective physiological roles *in vivo*.

### Distinct migration patterns emerge in bilayer spheroids of adult NSC niches

**Figure 2:**
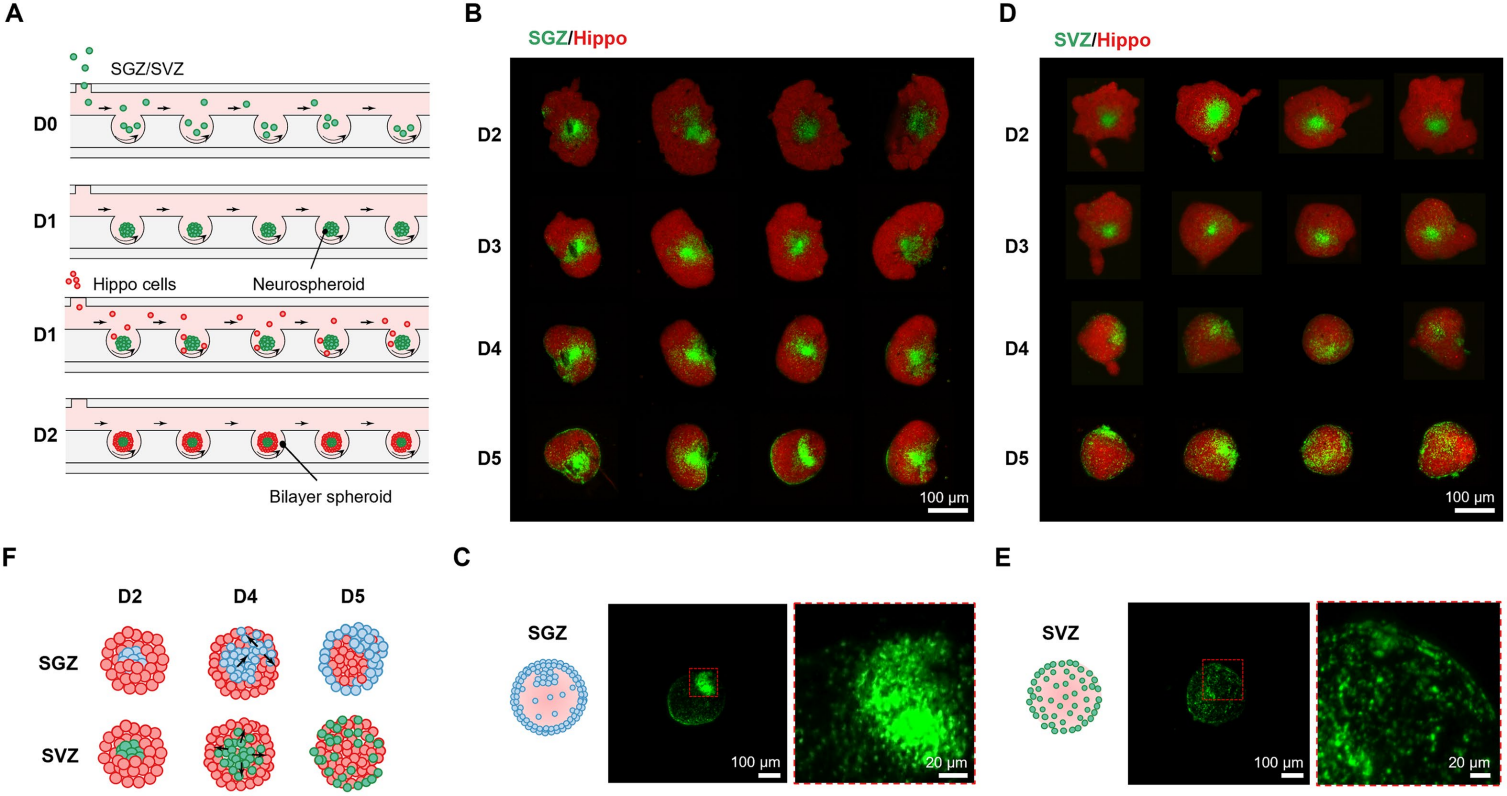
Distinct migration patterns of adult NSC-derived 3D bilayer spheroids. **(A)** Schematic illustration of neurospheroid and bilayer spheroid formation using a microfluidic chip under dynamic conditions. **(B,D)** Tracking of labeled SGZ- and SVZ-derived cells within bilayer spheroids from day 2 to day 5 (*n* = 5). Scale bar, 100 μm. **(C,E)** Representative images of SGZ- and SVZ-derived cells in bilayer spheroids. Red dashed boxes indicate magnified regions. Scale bars, 100 μm and 20 μm. **(F)** Schematic illustration of the migration direction of SGZ- and SVZ-derived cells within bilayer spheroids. NSCs, neural stem cells; SGZ, subgranular zone; SVZ, subventricular zone.

To better capture the three-dimensional (3D) interactions of adult NSCs with their microenvironment, we engineered bilayer spheroids under dynamic conditions by sequentially loading SGZ- or SVZ-derived cells and adult hippocampal neurons into a microfluidic chip (Figure 2A). By day 2 of coculture, both SGZ- and SVZ-derived cells established bilayer spheroids with hippocampal cell layers, but their migration dynamics diverged thereafter. In SGZ/hippocampal spheroids, SGZ-derived cells migrated into cohesive groups, often extending multicellular streams outward from the spheroid surface (Figures 2B,C). By contrast, in SVZ/hippocampal spheroids, SVZ-derived cells migrated in a more dispersed, individual manner, penetrating through the hippocampal cell layer as isolated cells (Figures 2D,E). These observations are qualitatively reminiscent of niche-associated migration patterns described *in vivo*, where SGZ neuroblasts display collective, radial migration toward the granule cell layer, whereas SVZ neuroblasts migrate individually or in chains toward distant targets (Figure 2F) (Lim and Alvarez-Buylla, 2016).

### Differential distribution of neuronal and glial populations within bilayer spheroids

**Figure 3:**
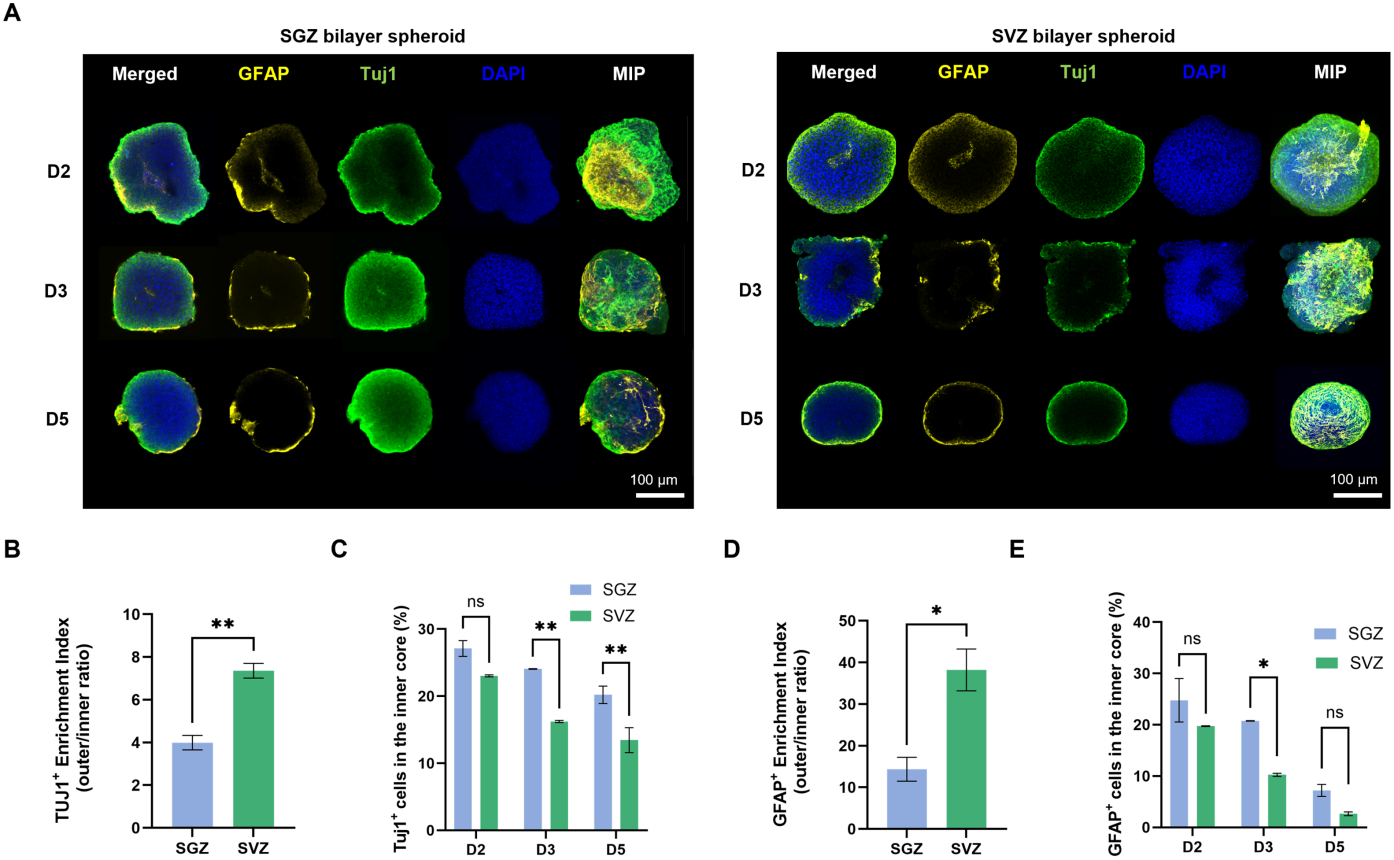
Differentiation and spatial distribution of SGZ- and SVZ-derived cells in 3D bilayer spheroids. **(A)** Representative immunofluorescence images of SGZ- and SVZ-derived bilayer spheroids at day 2, 3, and 5. Spheroids were stained for GFAP (yellow), TUJ1 (green), and nuclei (DAPI, blue). Images show the middle optical section and maximum intensity projection (MIP). Scale bar, 100 μm. **(B,D)** Enrichment index of TUJ1^+^ and GFAP^+^ cells on day 5, calculated as the ratio of outer-to-inner signal intensity, reflecting peripheral distribution of differentiated cells (*n* = 3 per group). **(C,E)** Quantification of the percentage of TUJ1^+^ and GFAP^+^ cells localized within the inner core of bilayer spheroids at different time points, normalized to total DAPI (*n* = 3 independent spheroids per time point). All data are presented as mean ± SEM. Statistical significance was determined using an unpaired two-tailed Student’s *t*-test for **(B,D)** and two-way ANOVA for **(C,E)**. ns, Nonsignificant; *p < 0.05, **p < 0.01, and ***p < 0.001. ANOVA, Analysis of Variance; DAPI, 4′,6-diamidino-2-phenylindole; MIP, maximum intensity projection; NSCs, neural stem cells; SEM, standard error of the mean; SGZ, subgranular zone; SVZ, subventricular zone.

We next investigated whether differences in migration were associated with lineage-specific differentiation patterns. Immunostaining for TUJ1 (neurons) and GFAP (astrocytes) was performed on bilayer spheroids at days 2, 3, and 5 (Figure 3A). Quantitative analysis revealed that SVZ-derived spheroids exhibited a significantly higher proportion of TUJ1^+^ and GFAP^+^ cells migrating outward into the spheroid periphery compared to SGZ-derived spheroids (Figures 3B,D). Conversely, SGZ-derived spheroids retained a greater proportion of both TUJ1^+^ and GFAP^+^ cells within the spheroid core (Figures 3C,E). Over time, SVZ-derived spheroids showed progressive neuronal dispersion, whereas SGZ-derived spheroids maintained core-restricted neuronal and glial populations. These data suggest that SVZ-derived NSCs are biased toward neuronal migration and integration at peripheral regions, whereas SGZ-derived NSCs maintain a mixed neuronal-glial identity concentrated within the core. This reflects known *in vivo* roles: SGZ-derived astrocytes contribute to local circuitry and niche maintenance, whereas SVZ-derived progenitors generate migratory neuroblasts that integrate into distal circuits (Ming and Song, 2011; Obernier and Alvarez-Buylla, 2019).

### Oligomeric oAβ_42_ induces morphological and targeted gene-expression alterations in DG–hippocampal bilayer spheroids

**Figure 4:**
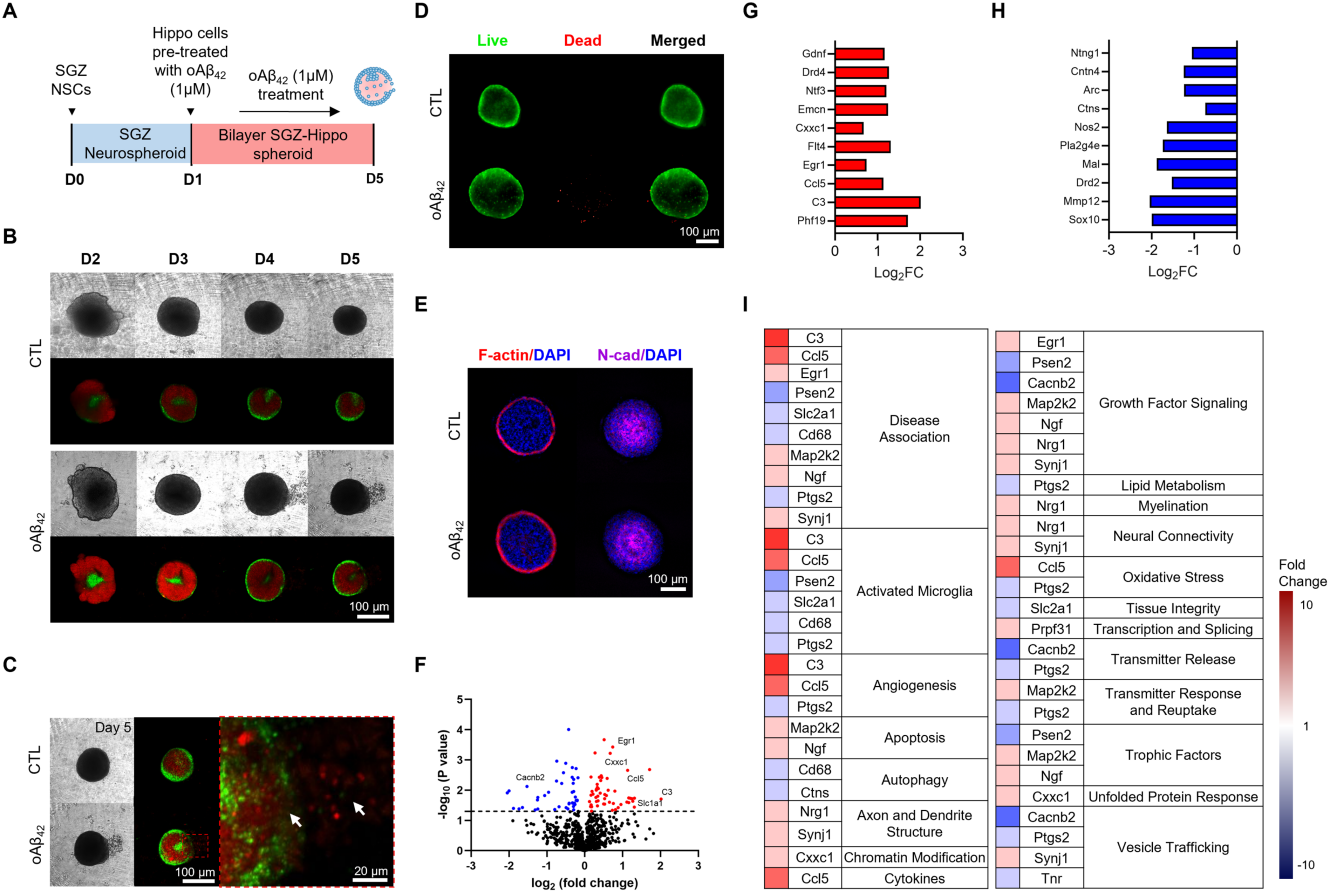
Morphological and targeted gene-expression alterations in SGZ–hippocampal bilayer spheroids following oligomer amyloid *β* (oAβ_42,_ 1 μM) exposure. **(A)** Schematic illustration of the experimental workflow for oAβ_42_ treatment of bilayer spheroids. **(B)** Phase-contrast and fluorescence images showing morphological changes in bilayer spheroids from day 2 to 5. SGZ-derived cells are shown in green and hippocampal cells in red. Scale bar, 100 μm. **(C)** Fluorescence images of bilayer spheroids on day 5. The red dashed box indicates magnified regions highlighting disrupted peripheral areas (white arrows) in oAβ42-treated spheroids compared with controls. Scale bars, 100 μm and 20 μm. **(D)** Fluorescence images of the live/dead assay performed on day 5 bilayer spheroids. Live cells are shown in green and dead cells in red. Scale bar, 100 μm. **(E)** Immunofluorescence staining of cytoskeletal and adhesion markers at day 5. F-actin (red) and N-cadherin (magenta) are shown with DAPI (blue). Scale bar, 100 μm. **(F)** Volcano plot of differentially expressed genes in oAβ-treated spheroids on day 5. The dashed line indicates the significance threshold (*p* = 0.05). **(G,H)** Bar plots showing representative significantly upregulated **(G)** and downregulated **(H)** genes. **(I)** Heatmap of differentially expressed genes grouped by functional categories, including inflammatory response, microglial activation, angiogenesis, apoptosis, synaptic function, and growth-factor signaling. Aβ, amyloid-β; ANOVA, analysis of variance; DAPI, 4′,6-diamidino-2-phenylindole; DG, dentate gyrus; MIP, maximum intensity projection; NSCs, neural stem cells; oAβ42, oligomeric amyloid-β42; SEM, standard error of the mean; SGZ, subgranular zone; SVZ, subventricular zone.

Next, we took advantage of our adult neurogenesis niche model to examine Aβ pathology. SGZ bilayer spheroids were treated with oAβ_42_ (1 μM) for 4 days (Figure 4A). Morphologically, oAβ_42_-treated bilayer spheroids exhibited fragmented outer layers compared to the compact structure observed in untreated controls over the same period (Figures 4B,C; Supplementary Figure 5). Qualitative inspection suggested that bilayer formation and collective SGZ migration patterns appeared to be maintained in the presence of oAβ_42_-pretreated hippocampal cells, although quantitative migration tracking was not performed under this condition (Figure 4B). From day 4 to 5, oAβ_42_-exposed spheroids demonstrated progressive cell debris emerging at the spheroid edges (Figures 4B,C). A live/dead assay further revealed increased cell death in oAβ_42_-treated spheroids compared to controls, indicating cytotoxic effects (Figure 4D).

To visualize cytoskeletal organization and cell–cell adhesion patterns, spheroids were stained for F-actin and N-cadherin (Figure 4E). In control spheroids, F-actin staining appeared relatively organized along the spheroid periphery. In contrast, oAβ_42_-treated spheroids showed a thicker and more irregular F-actin staining pattern, suggesting altered cytoskeletal organization. N-cadherin staining was detectable in both control and oAβ_42_-treated spheroids. However, treated spheroids appeared to show a more heterogeneous staining pattern, with relatively weaker central signal compared with controls. Together with the live/dead staining results, these findings suggest that oAβ_42_ exposure is associated with increased cell death and disruption of spheroid organization.

Targeted gene expression profiling using the NanoString nCounter Mouse Neuropathology Panel revealed significant gene-expression changes following oAβ_42_ exposure (Figure 4F). The volcano plot demonstrated significant upregulation of *C3*, *Ccl5*, and *Cxcl1*, markers of acute inflammatory response, alongside downregulation of *Cacnb2*, a gene associated with calcium-channel function (Figures 4G,H). Among the upregulated genes, *C3* exhibited the highest fold-change expression, consistent with activation of complement-mediated inflammatory pathways (Figure 4G). Conversely, downregulated genes included synaptic- and calcium-channel-associated transcripts such as *Cacnb2*, *Arc*, and *Cntn4*, suggesting compromised neuronal signaling capacity (Figure 4H). Gene ontology analysis further highlighted enrichment in pathways linked to neuroinflammation, activated microglia, apoptosis, and reduced synaptic signaling (Figure 4I). Collectively, these findings suggest that oAβ_42_ exposure is associated with structural and targeted gene-expression alterations in SGZ-derived bilayer spheroids.

## Discussion

Our study demonstrates that adult NSCs derived from distinct neurogenic niches exhibit different morphological, migratory, and differentiation behaviors when cultured in engineered 2D and 3D microenvironments. Specifically, SVZ-derived progenitors displayed greater motility, extended neurite outgrowth, and a dispersed migration pattern, whereas SGZ-derived progenitors migrated more collectively with a core-restricted, mixed neuronal-glial distribution. These findings are consistent with niche-associated migration patterns of adult neurogenesis at the cellular level.

Previous studies have largely characterized adult neurogenesis and niche-specific differences using histological or *in vivo* imaging approaches (Doetsch et al., 1999; Ming and Song, 2011). While these studies revealed broad differences between SGZ and SVZ progenitors, *in vitro* assays have been limited in capturing unique migration dynamics. Conventional neurosphere assays, for instance, primarily assess proliferative capacity and multipotency (Reynolds and Weiss, 1992) but fail to recapitulate migration, whereas 2D cultures lack the spatial complexity of *in vivo* microenvironments. To address this limitation, we established a migration chip that enables real-time quantification of single-cell motility (Figure 1) and a 3D bilayer spheroid platform that allows observation of the migration and behavior of SGZ- and SVZ-derived NSCs within the outer adult cells scaffold (Figures 2–4).

A key advantage of this approach lies in its ability to reveal collective versus individual migration behaviors. In the migration chip (Figures 1G–I), SVZ-derived cells showed greater migration distances relative to SGZ-derived cells, although we acknowledge that this time-course experiment was performed with a single chip and that independent biological replicates will be required to confirm the reproducibility of the observed motility differences. Notwithstanding this limitation, the differences in motility are broadly consistent with *in vivo* patterns, where SGZ neuroblasts exhibit localized radial migration and SVZ neuroblasts undergo long-range tangential migration (Lois and Alvarez-Buylla, 1994; Bond et al., 2015).

Within bilayer spheroids, SGZ-derived cells maintained compact rim migration and a dense inner core, whereas SVZ-derived cells dispersed more broadly across the outer layer (Figure 2). This spatial organization is linked to lineage specification and directional migration, underscoring the contribution of both cell-intrinsic properties and extrinsic niche signals. Notably, our bilayer spheroid extends conventional 3D approaches by intentionally recreating a discrete inner stem/progenitor core within an outer adult-cell scaffold, allowing direct comparison of how the external cellular environment influences distinct progenitor populations. This bilayer organization provides structural guidance and a defined microenvironment that may elicit physiologically relevant migratory behaviors.

Our findings revealed pronounced niche-specific differences in neuroglial organization within bilayer spheroids (Figure 3). Quantitative analysis of inner-core versus outer-region distribution revealed that SGZ-derived spheroids retained a higher proportion of TUJ1^+^ neurons and GFAP^+^ astrocytes within the inner core across days 2–5, reflecting the compact, radial migration pattern of the DG *in vivo*. In contrast, SVZ-derived spheroids exhibited peripheral enrichment of both neuronal and glial cells, consistent with the tangential dispersal characteristic of the rostral migratory stream. Temporal dynamics further demonstrated sustained inner-core retention in SGZ spheroids, whereas SVZ progeny progressively migrated outward. These results are consistent with *in vivo* niche behaviors (Doetsch et al., 1999; Ming and Song, 2011; Fuentealba et al., 2012; Obernier and Alvarez-Buylla, 2019) and support the use of the bilayer spheroid system as a relevant *in vitro* platform for studying adult NSC lineage distribution and migration.

Neuronal migration is regulated through the complex activities of intrinsic and extrinsic niche-derived signals. Known regulators such as Rho signaling, Rapgef2, and ephrin/Eph signaling have been shown to orchestrate NSC migration *in vivo* (Ota et al., 2014; Ye et al., 2014; Todd et al., 2017). Our observation that migration patterns persisted across both 2D and 3D environments, whether or not hippocampal cues were present, suggests that NSC behavior is driven by a combination of intrinsic properties and extrinsic guidance signals. Future studies could specifically dissect these contributions by selectively perturbing known molecular pathways in our migration platforms.

Oligomer Aβ (1 μM) exposure was associated with morphological and targeted gene-expression alterations in DG–hippocampal bilayer spheroids, reflecting selected features of Aβ-associated neurotoxicity (Figure 4). Aβ-treated spheroids displayed more irregular borders and reduced compactness, and live/dead, F-actin, and N-cadherin imaging suggested increased cell death and altered spheroid organization. Although gross SGZ migration morphology remained visible after oAβ42 exposure, migration parameters were not quantitatively measured under this condition. Therefore, further quantitative analysis will be needed to determine how Aβ exposure affects SGZ cell dynamics and spheroid organization in this model.

Targeted gene-expression profiling revealed a strong induction of inflammatory- and immune-related genes such as *C3* and *Ccl5*, alongside the downregulation of *Cacnb2*, a calcium-channel subunit crucial for synaptic transmission. Gene ontology and pathway analysis further indicated enrichment in neuroinflammatory- and apoptosis-related pathways, including activated microglia, oxidative stress, and unfolded protein response, whereas genes linked to synaptic signaling and myelination were suppressed. These molecular changes are consistent with prior findings that Aβ oligomers trigger complement activation and chemokine-mediated neuroinflammation, contributing to synaptic loss and neuronal dysfunction (Tu et al., 2014; Wu et al., 2019). Moreover, although our model utilizes adult mouse NSCs, the Aβ-induced gene-expression changes observed in our bilayer spheroids, including complement activation and synaptic suppression, partially overlap with transcriptomic signatures reported in human AD brain cohorts, supporting the translational relevance of our SGZ–hippocampal bilayer platform (Peng et al., 2021). Collectively, these results demonstrate that the bilayer hippocampal spheroid system can reflect selected pathological features associated with Aβ-induced neurotoxicity and offers a relevant platform for investigating AD-related mechanisms within the adult neurogenesis niche.

Moreover, adult neurogenic niches differ in several key aspects, including their distribution of neuroglial lineages, rates of cell migration, and regional integration targets (Fuentealba et al., 2012; Kaneko et al., 2017; Obernier and Alvarez-Buylla, 2019). While iPSC-based systems have expanded access to human NSCs, they provide limited insights into adult-specific processes. The use of adult mouse NSCs in our model captures niche-associated dynamics that may be altered during aging, an area that warrants further investigation.

Despite its advantages, several limitations should be considered. While baseline characterization of NSCs and hippocampal cells was performed (Supplementary Figure 1), further detailed phenotypic profiling may help refine the interpretation of lineage-specific behaviors. Moreover, the bilayer spheroid model lacks vascular, immune, and extracellular matrix (ECM) components, which are essential regulators of the NSC niche *in vivo* (Goldman and Chen, 2011). In particular, vascular-derived cues such as vascular endothelial growth factor, immune-derived cytokines, and ECM proteins such as laminin and tenascin-C are known regulators of adult neurogenesis (Joo et al., 2015; Tucić et al., 2021). Incorporating vascular and glial cocultures, as well as matrix engineering, into our migration chip and bilayer models will further enhance the physiological relevance of the neurogenic niche. Furthermore, immunogenic profiling of NSCs was not assessed in this study. While immunogenicity is an important consideration for therapeutic applications, future studies incorporating immune-related components such as microglia, peripheral immune cells, and astrocyte-mediated inflammatory signaling would be beneficial. Importantly, migration in this system reflects intrinsic motility under compartmentalized conditions rather than a defined chemotactic gradient. The observed differences may arise from biophysical or metabolic properties, such as nuclear deformability or cytoskeletal organization, or responses to glucose- and lactate-rich environments, although these parameters were not directly evaluated in the present study. Future studies employing microchannel chip assays may help to further elucidate these mechanisms.

In conclusion, our study highlights intrinsic niche-dependent differences in the migration and lineage organization of adult NSCs and establishes versatile *in vitro* platforms for their comparative analysis. By enabling the investigation of NSC behavior in both microchannel systems and 3D layered culture microenvironments, these approaches provide a framework for mechanistic studies of adult neurogenesis and offer potential applications in disease modeling and regenerative medicine.

## Data Availability

The original contributions presented in the study are included in the article/Supplementary material, further inquiries can be directed to the corresponding author.
