# Supplementary material for: Distinct migration patterns of adult neural stem cells derived from hippocampal and ventricular niches
Source: Front Aging Neurosci. 2026 Jun 3;18:1727458. doi: 10.3389/fnagi.2026.1727458 (PMC13272391; doi:10.3389/fnagi.2026.1727458)
Supplement: Supplementary file 1 [file Data_Sheet_1.PDF]

Distinct Migration Patterns of Adult Neural Stem Cells Derived from Hippocampal and Ventricular niches

Sarnai Amartumur[1†], Huong T.L. Nguyen[1†], Trung Hoang[1], Thuy Huynh[1], Chaejeong Heo[1]*

[1]Department of Biophysics, Institute of Quantum Biophysics, Sungkyunkwan University, Suwon 16419, Republic of Korea

List of Supplementary Figures:

Supplementary Figure 01 | Characterization of adult SGZ- and SVZ-derived NSCs and adult hippocampal neuron

Supplementary Figure 02 | Morphology difference in **(A)** DG vs **(B)** SVZ-derived differentiation cells at day 2

Supplementary Figure 03 | Migration difference in DG vs SVZ-derived differentiation cells at day 1

Supplementary Figure 04 | Microfluidic chip designs and culture system

Supplementary Figure 05 | Migration of SVZ, SGZ-derived cells after 6 hours through the microchannels

Supplementary Figures

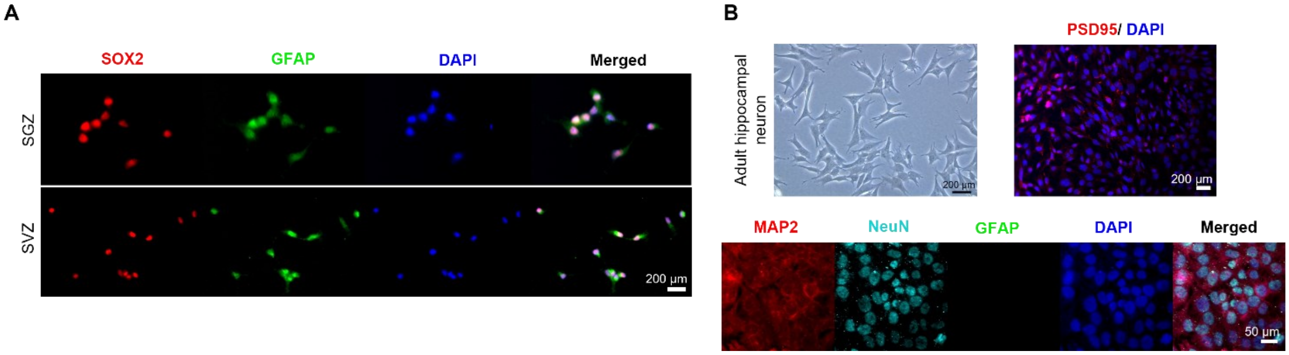

Supplementary Figure 01 | Characterization of adult SGZ- and SVZ-derived NSCs and adult hippocampal neuron. (**A**) Representative fluorescence images of SGZ- and SVZ-derived NSCs. Cells were stained for SOX2 (red), and GFAP (green), with nuclear counterstaining by DAPI (blue). Scale bar, 200 µm. (**B)** Phase-contrast and fluorescence images of adult mouse hippocampal neurons. Cells were stained for PSD95 (red), MAP2 (red), and NeuN (cyan), and GFAP (green). Nuclei are counterstained with DAPI (blue). Scale bars, 200 µm (top panels) and 50 µm (bottom panels).

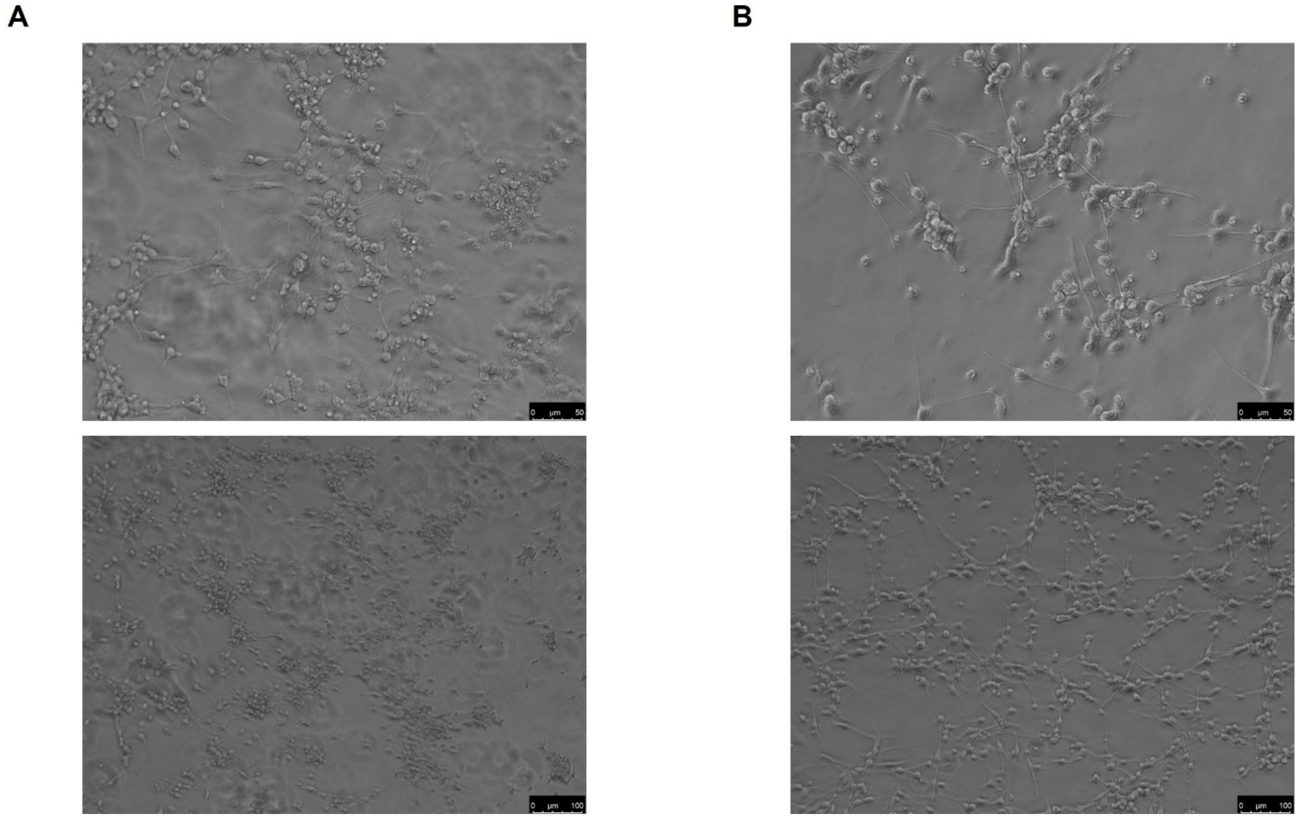

Supplementary Figure 02 | Morphology difference in **(A)** SGZ vs **(B)** SVZ-derived cells in differentiation media at day 2. (n=4 per group). Two different images represent for the SGZ in A and SVZ in B. Scale bars, 50 µm (top), 100 µm (bottom).

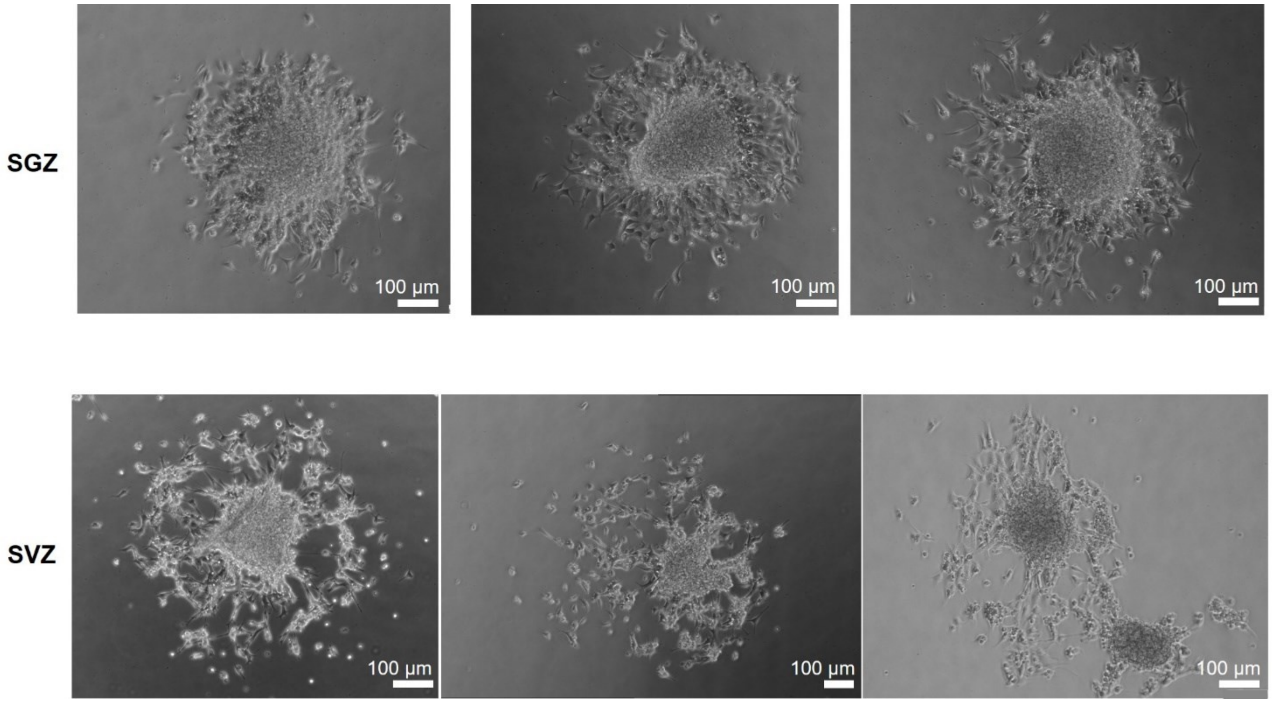

Supplementary Figure 03 | Migration difference in SGZ and SVZ-derived differentiation cells at day 1 (n=5 per group). Three different images of migrated neurospheres represent for SGZ and SVZ. Scale bar, 100 µm.

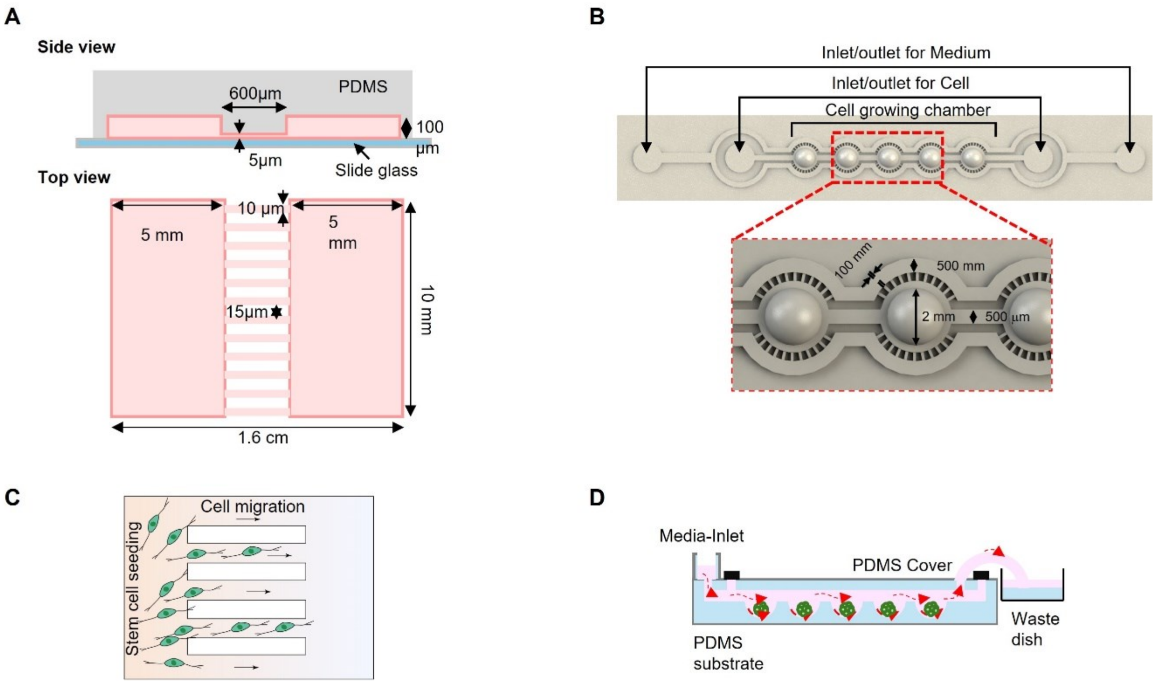

Supplementary Figure 04 | Microfluidic chip designs and culture system**. (A)** Schematic illustration of the migration chip geometry. **(B)** Image of the microfluidic chip mold indicating medium inlets/outlets, cell inlets/outlets, and the cell-growing chamber. Magnified view highlights the spheroid culture chambers (2 mm diameter). **(C)** Schematic representation of the migration assay. Stem cells are seeded on one side of the chamber and migrate through confined parallel microchannels toward the opposite compartment. (**D)** Cross-sectional schematic of the spheroid culturing system illustrating gravity-driven medium flow from the media inlet toward the waste reservoir, maintaining compartmentalized culture conditions.

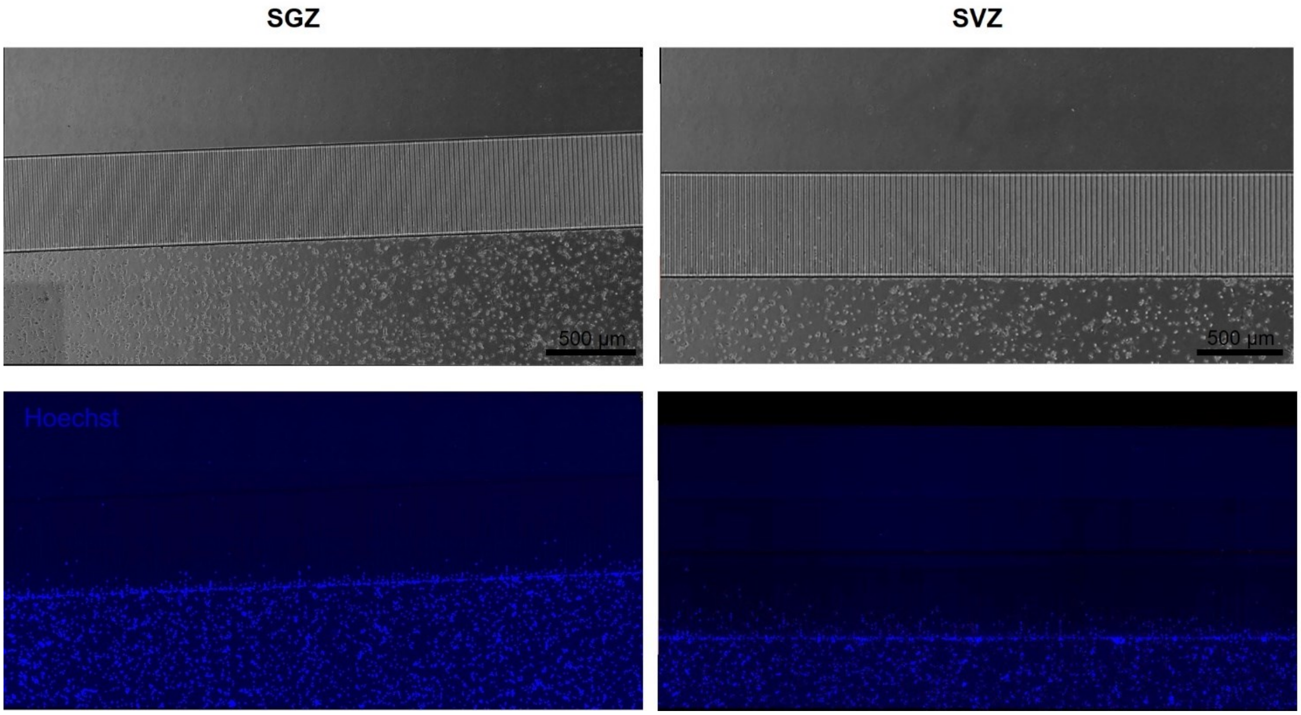

Supplementary Figure 05 | Migration of SVZ, SGZ-derived cells after 6 hours through the microchannels. Top panels show phase-contrast images, and bottom panels show nuclei stained with Hoechst. (n=1 chip per group). Scale bars, 500 μm.
